# A 16-week aerobic exercise and mindfulness-based intervention on chronic psychosocial stress: a pilot and feasibility study

**DOI:** 10.1186/s40814-020-00751-6

**Published:** 2021-03-06

**Authors:** Guy A. Prochilo, Ricardo J.S. Costa, Craig Hassed, Richard Chambers, Pascal Molenberghs

**Affiliations:** 1 Melbourne School of Psychological Sciences, University of Melbourne, Melbourne, Australia; 2 ISN Psychology, Institute for Social Neuroscience, Melbourne, Australia; 3 Department of Nutrition Dietetics & Food, Monash University, Melbourne, Australia; 4 Department of General Practice, Monash University, Melbourne, Australia; 5 Mindfulness Programs, Monash University, Melbourne, Australia

**Keywords:** Pilot and feasibility study, Aerobic exercise, Mindfulness, Mental and physical training, Nonclinical sample

## Abstract

**Objectives:**

Researchers have begun delivering mindfulness and aerobic exercise training concurrently on the premise that a combination intervention will yield salutary outcomes over and above each intervention alone. An estimate of the effect of combination training on chronic psychosocial stress in a nonclinical population has not been established. The objective of this study was to establish protocol feasibility in preparation of a definitive RCT targeting healthy individuals, and to explore the preliminary effect of combination training on reducing chronic psychosocial stress in this population.

**Methods:**

Twenty-four participants were allocated to a single-arm pre-post study and subjected to 16 weeks of concurrent mindfulness psychoeducation and aerobic exercise training. Feasibility criteria were collected and evaluated. Within-group changes in chronic psychosocial stress, mindfulness, emotion regulation, and cardiorespiratory fitness were also assessed. Primary analyses were based on 17 participants.

**Results:**

Retention rate, response rate, recruitment rate, and sample size analyses indicate a definitive trial is feasible for detecting most effects with precision. There was also a decline in our primary dependent measure of chronic psychosocial stress (*d*_*pretest*_ = −0.56, 95% CI [ −1.14,−0.06]). With regard to secondary measures, there was an increase in the use of cognitive reappraisal, and a reduction in use of maladaptive emotion regulation strategies. We are insufficiently confident to comment on changes in mindfulness and aerobic capacity $\left (\dot {V}O_{2max}\right)$. However, there were subgroup improvements in aerobic economy at submaximal exercise intensities.

**Conclusions:**

We recommend a definitive trial is feasible and should proceed.

**Trial registration:**

ANZCTR (ID: ACTRN12619001726145). Retrospectively registered December 9, 2019.

**Supplementary Information:**

The online version contains supplementary material available at (10.1186/s40814-020-00751-6).

## Background

A growing body of research has implicated psychosocial stress with a range of deleterious health outcomes. At an individual level, this includes a greater incidence of depressive symptoms, anxiety, and a decline in wellbeing [1, 2], as well as impairments of physical health [3, 4]. To address these problems, interventions involving aerobic exercise [5] and mindfulness [6] have been found effective for improving mental health outcomes. Given these benefits, researchers have also begun delivering these interventions concurrently [7]. The rationale for a combination training modality is that such training may yield salutary outcomes over and above each intervention alone [8].

Few studies have examined the effect of combining aerobic exercise and mindfulness-based training on mental health outcomes. To our knowledge, an estimate of the range and direction of the effect of a combination intervention on chronic psychosocial stress in a nonclinical population, specifically, is also absent from the literature. This pilot and feasibility study was conducted to guide future research on combination training with respect to its effect on chronic psychosocial stress, as well as its potential mechanisms. To this end, we conducted a single-arm pre-post study in preparation for a future randomized controlled trial (RCT). This study comprised an aerobic exercise and mindfulness-based intervention delivered concurrently over 16 weeks, and was designed as a prevention-focused intervention to be used by healthy individuals rather than those presenting with specific mental health risk factors. In the following sections, we discuss the evidence and theorized mechanisms that link mindfulness, aerobic exercise, and combination training to salutary mental health outcomes. We conclude with our study rationale.

## Mindfulness

Mindfulness is a mental practice that draws on contemplative traditions and evidence-based research, and which has been adopted as a therapeutic approach to promote mental and physical health [9]. In a clinical and research context, mindfulness has been described as a non-judgmental, non-reactive, and non-elaborative attention to the present moment experience, and is an innate capacity that can be strengthened through formal meditation and informal practice [10]. Mindfulness-based interventions (MBIs) have proliferated, and include Mindfulness-based Stress Reduction [11] and Mindfulness-based Cognitive Therapy [12], among others. MBIs vary widely in their focus, content, and structure. However, the formal features that define an MBI are inclusion of (1) systematic and sustained training in formal meditation and informal practices, and (2) an underlying theoretical model based around training the attention to spend more time in the present moment [13].

Meta-analysis of controlled trials suggests that MBIs may improve multiple indices of mental health (here reported as the between-group standardized mean difference, *d*, reported in each publication). This includes a reduction in psychosocial stress (*d* = −0.74, 95% CI [ −1.07,−.41]), anxiety (*d* = −0.64, 95% CI [ −.94,−.33]), and depressive symptoms (*d* = −0.80, 95% CI [ −1.12,−.49]) [6]. There also appears to be a dose-response relationship between formal meditation practice and salutary mental health outcomes (*r* =.26) [14]. However, the relationship between the overall duration of an MBI and these outcomes remains equivocal [6].

The cognitive and emotional mechanisms by which MBIs exert their effects on mental health have been assessed through a meta-analysis of intervention studies using mediation models [15]. These authors applied a best evidence synthesis rating system (BESRS) to determine if a body of evidence was considered strong, moderate, or insufficient [16]. They reported strong evidence for reduced cognitive and emotional reactivity, and moderate evidence for dispositional mindfulness and repetitive negative thinking as predictors of improvement in mental health (pooled correlations: *r*s =.33 to.36). These mechanisms are additionally captured by mindfulness theories. For example, the mindfulness-to-meaning theory suggests that mindfulness may help individuals select more adaptive emotion regulation strategies in times of stress, and thereby reduce reactivity to stressful events [17]. Particular interest is given to cognitive reappraisal, which is a strategy that involves actively reinterpreting emotional stimuli to modify its emotion impact. Cognitive reappraisal is considered a central mechanism in some mindfulness theories [17], although is a secondary effect in others [18].

## Aerobic exercise

Physical activity is a broad term that applies to any bodily movement or activity produced by skeletal muscles that require energy expenditure [19]. Aerobic exercise is a specific subcategory of physical activity that is characterized by rhythmic, sustained movement of large muscle groups, and which is primarily dependent on energy-generating processes that occur through oxygen metabolism. The primary goal of aerobic exercise is to improve or maintain cardiorespiratory fitness, which is the capacity of the circulatory and respiratory system to supply oxygen during sustained exercise.

Aerobic exercise is the most widely examined exercise modality with respect to mental health outcomes, and there is sufficient evidence to suggest it is implicated in improvements in psychological health. For example, meta-analyses of RCT interventions show that aerobic exercises of various modalities are capable of reducing depressive symptoms (*d* = −0.55, 95% CI [ −0.77,−0.34]) [5] and anxiety symptoms (*d* = −0.58, 95% CI [ −1.0 to −0.76]) [20]. There is also meta-analytic evidence that the effect of aerobic exercise on mental health outcomes may follow a dose-response relationship with the number of exercise sessions completed (although effect size may decline in magnitude at very high frequencies, e.g., 37+ exercise sessions) [5]. Meanwhile, the relationship between effect size and intensity of exercise has not yet been established [5, 21].

Aerobic exercise can be implicated in improvement in cardiorespiratory fitness, which in turn is associated with widespread physiological effects that enhance cardiovascular health. For example, this includes improved glucose tolerance, reduced low-grade chronic inflammation, and a lower prevalence of hypertension [22]. With respect to psychosocial stress, it has been hypothesized that the salutary mental health outcomes that arise following exercise training may be related to exercise-associated adaptations to stress-responsive systems [23]. This includes a reduction in reactivity of cardiovascular, metabolic, autonomic, and neuroendocrine systems in response to exercise stress, which carry over to a reduction in reactivity in response to psychosocial stressors. Consequently, cardiorespiratory fitness may be an important mediator of improvements in mental health outcomes observed following regular aerobic exercise.

Self-reports of physical activity are a subjective construct that measure behaviors that may be associated with cardiorespiratory fitness. These constructs are often implicated in a reduction in psychosocial stress and the prevalence of stress-related disorders [24, 25]. However, direct assessments of cardiorespiratory fitness provide a more objective method of investigating the relationship between fitness and mental health.

In the exercise physiology literature, two objective measures include maximal aerobic capacity $\left (\dot {V}O_{2max}\right)$ and aerobic economy [26, 27]. $\dot {V}O_{2max}$ represents the maximal amount of oxygen that can be consumed during exhaustive exercise, where a higher $\dot {V}O_{2max}$ is indicative of greater cardiorespiratory fitness. Aerobic economy represents the steady-state energy requirement of exercise conducted at a constant exercise intensity (e.g., running at a constant velocity). Here, a lower energy requirement at a constant intensity is indicative of higher aerobic economy and greater cardiorespiratory fitness. Research examining the relationship between $\dot {V}O_{2max}$ and mental health outcomes has so far been mixed. Several cross-sectional studies have reported no relationship between $\dot {V}O_{2max}$ and mental health [28, 29], while a recent controlled trial reported that an improvement in $\dot {V}O_{2max}$ was associated with a reduction in psychosocial stress [30]. Meanwhile, aerobic economy is relatively understudied in the mental health literature altogether.

## Combination training

Improvements in mental health outcomes are often observed in interventions that make use of mindfulness in combination with physical activity [11]. One recent example is Mindful2Work: a single-arm pilot and feasibility study that delivered mindfulness, physical activity, and yoga training concurrently to a sample of employees medically diagnosed with severe work-related stress [31]. This study comprised mindfulness psychoeducation (80 min/week), home mindfulness practice (20 min/day), physical activity (20 min twice per week), and yoga exercises (10 min twice per week) completed concurrently over a period of 6 weeks. The authors reported that participants declined in chronic psychosocial stress and stress-related symptoms, and that these effects were also maintained at a 6-week follow-up.

Unfortunately, such studies tend to examine physical activity broadly, rather than through structured aerobic exercise conducted at a frequency and intensity sufficient to maintain or improve cardiorespiratory fitness. For example, in the above study, physical activity comprised a combination of (unspecified) aerobic and strength exercises. The authors also relied on a subjective measure of intensity (i.e., 70% of one’s full capacity) rather than objective assessments via $\dot {V}O_{2max}$.

One exception is mental and physical (MAP) training: an intervention involving focused-attention meditation in combination with aerobic exercise [7] (mode = treadmill running or stationary cycling; intensity = heart rate equivalent to 50–70% $\dot {V}O_{2peak}$). Mental and physical training components in this program were each conducted in separate 30-min sessions twice per week for 8 weeks. The main outcomes of this study focused on pre-post changes in depressive symptoms and the disposition to ruminate separately in a group of clinically depressed and non-depressed participants. In this study, it was shown that MAP training was successful in improving each of these mental health outcomes in both groups, but decreases in depressive symptoms were greater within the depressed group than the non-depressed group. Moreover, there were no reported changes in cardiorespiratory fitness for any group of participants.

## Rationale for a pilot and feasibility study

The above studies provide preliminary support that combining aerobic exercise and mindfulness-based training may improve mental health outcomes. However, these studies fall short of informing the development of a future RCT as the basis for a prevention-focused stress reduction intervention in a nonclinical population. To this end, we recruited a sample of healthy adults and conducted a single-arm pre-post pilot and feasibility study that comprised an aerobic exercise and mindfulness-based intervention that were completed concurrently over 16 weeks. The aerobic exercise component, specifically, comprised a mixture of endurance running and interval training completed at a frequency and intensity considered sufficient to maintain or improve cardiorespiratory fitness [32]. All comparisons performed in this study were pre-to-post assessments. The primary research outcomes were twofold. First, we aimed to obtain sufficient assurance of protocol feasibility in a healthy population with respect to retention rate, assessment response rate, recruitment rate, and required sample size for a confirmatory trial. And second, we aimed to obtain a preliminary estimate of within-group changes in chronic psychosocial stress in this population, as well as an estimate of change in secondary outcomes that may explain changes in chronic psychosocial stress (i.e, mindfulness, emotion regulation factors, and cardiorespiratory fitness).

## Methods

### Study design

This pilot and feasibility study was conducted as a single-arm pre-post study where all participants were subjected to aerobic exercise and mindfulness-based training, delivered concurrently. Assuming that the relationship between intervention exposure and its effect on psychosocial stress is linear, there will be greater statistical power to detect within-group changes as the intervention duration increases. Therefore, in this study, we doubled the typical duration of a mindfulness or MAP intervention and subjected participants to 16 weeks of training.

This study was conducted across two waves: wave 1 (August to November 2016, *n* = 5) and wave 2 (April to July 2017, *n* = 19). Recruitment was conducted across a 1-month time period prior to the intervention period of each wave. Pre-test (T0) and post-test (T1) measures were obtained 1 to 4 weeks prior to and after the intervention period. Participants were predominantly postgraduate and undergraduate students from a variety of disciplines, and these periods were selected to correspond to low-stress periods in the academic calendar (i.e., pre- or post-semester). Responses to self-report measures were collected online via Qualtrics (www.qualtrics.com), and assessments of cardiorespiratory fitness were collected in an exercise laboratory at Monash University. Descriptions of each assessment are provided in the “Methods” section below. No remuneration was offered.

### Participants

Participants self-selected into the study by responding to an advertisement posted to online community groups based in Melbourne, Australia. These advertisements described the study as a mental and physical training program for stress management. Young adults between the ages of 18 and 35 years were targeted to reduce age-related variance in assessment responses, and because this age group was considered less likely to experience an adverse exercise-related incident. Exclusion criteria were as follows: (1) age not within 18–35 years, (2) prior completion of an endurance event equivalent to a half-marathon (21.1 km), (3) prior completion of a formal meditation program, (4) current diagnosis of a neurological or mental disorder, (5) current diagnosis/history of chronic pain or musculoskeletal conditions, (6) current diagnosis/history of chronic disease of any kind, (7) current diagnosis of a heat or cold disorder, (8) current use of medication that influences the neuroendocrine or immune system, (9) current injury of any kind (e.g., joint or muscle injury), (10) current diagnosis of an infectious disease, (11) BMI ≥ 30 kg/m^2^, or (12) pregnancy or suspected pregnancy. All participants reported no engagement in regular running training or meditation practice within 6 months prior to the first assessment.

Pilot study methodologists suggest that 15–20 participants per cell of a pilot research design can provide a reasonable estimate for most medium to large standardized effects as defined by Cohen [33], without wasting resources [34]. To attain at least 15 participants and account for an estimated dropout rate of 30–35%, the rule for termination of data collection was set between 24 and 26 participants.

### Intervention

The training program involved a mindfulness-based intervention (MBI) and aerobic exercise training program completed concurrently over a period of 16 weeks. Each component of the program is described in detail in Supplementary Materials.

Briefly, the MBI comprised eight group psychoeducation and reflection activity sessions (55 min each) and formal focused-attention meditation practice (up to 20 min of individual home practice, conducted daily). All group training sessions followed a similar structure (Supplementary Materials Table 1) and were interspersed throughout the full 16-week program (Supplementary Materials Table 2).

The exercise program comprised three runs per week over 16 weeks following a half-marathon training schedule (Supplementary Materials Table 3), alongside several training support workshops. The exercise program involved a combination of high-intensity interval training and moderate-to-vigorous intensity endurance training, with the latter gradually increasing in duration across the intervention. Exercise intensity categories assigned to each run were formally defined by the ACSM exercise prescription guidelines [32]. Participants differed in cardiorespiratory fitness at baseline as identified through $\dot {V}O_{2max}$ testing. Therefore, exercise prescriptions adhering to these categories (i.e., the velocity required to attain a specific intensity category) were individualized to each participant by plotting oxygen consumption ($\dot {V}O_{2}$ mL/kg/min) against running velocity (km/h) in a regression model derived from the baseline $\dot {V}O_{2max}$ test. Participants were issued a GPS-enabled sportswatch (Garmin Forerunner 235, Garmin, USA) to guide and track their performance.

Adherence requirements for the MBI were that participants attend at least 50% of the group psychoeducation sessions and complete at least 50% of the formal meditation target (target = 37.34 h 20 min/day over 16 weeks; adherence requirement = 18.67 h). Compliance was monitored through attendance records and self-report of formal practice in an online spreadsheet (Google Sheets, Google, USA). Adherence requirements for the exercise program were that participants complete at least 50% of all prescribed running sessions (target = 48 individual runs 3 runs/week over 16 weeks; adherence requirement = 24 runs). Additional measures of dosage included total running distance (km), total running time (h), and mean exercise intensity (reported as the mean percentage of $\dot {V}O_{2max}$ at which a participant conducted their training averaged across all running sessions). Compliance was monitored through GPS-enabled sportswatch data (i.e., running time, velocity, and distance) which was recorded for each running session and uploaded to an online database (http://connect.garmin.com).

## Measures

### Protocol feasibility

#### Retention rate

This measure was quantified as the percentage of participants allocated into this study who successfully completed the study. A recent meta-analysis of MBIs for healthy (i.e., nonclinical) individuals reported a mean dropout rate of 17.0% [6]. However, this dropout rate ranged from 3.0 to 34.9% (65.1–97.0% retention) across studies. With respect to aerobic exercise interventions, attrition rates in previously non-exercising individuals have been reported to range from 7 to 58% [35]. Based on this information, we determined that a retention rate as low as 65% was an acceptable criterion to recommend proceeding with a definitive trial. This corresponded to the lowest retention rate reported for MBIs in healthy individuals. Because MBIs typically have a high retention and have been shown effective for reducing chronic psychosocial stress, a retention lower than 65% may indicate that this intervention is not feasible compared to already existing effective interventions.

#### Assessment response rate

This measure was quantified as the percentage of participants providing full pre-test (T0) and post-test (T1) data points across the study. An assessment response rate of 95% for our primary dependent variable (chronic psychosocial stress) was considered an acceptable criterion to recommend proceeding with a definitive trial.

#### Recruitment rate and sample size planning for a future definitive trial

Recruitment rate was quantified as the percentage of all applicants who satisfied the inclusion criteria and agreed to participate. The required sample size was computed for T1–T0 gain scores on the chronic psychosocial stress measure (within-group changes), as well as for hypothetical differences between independent arms of a confirmatory trial (between-group changes) [33]. Recruitment and sample size feasibility were assessed against a 5-year protocol where the 16-week program is repeated twice per year for a total of 10 trials. A further assumption was made that it would only be feasible to conduct each trial run with a total 20 participants per treatment arm (i.e., with four treatment arms: *n* = 80 per trial). This meant that the total maximum number of participants across 10 trials would be *n* = 200 per treatment arm (i.e., total *N* = 800).

### Estimation of participant-centered outcomes

#### Chronic psychosocial stress

The Perceived Stress Scale (PSS-10) [33] was the primary measure of chronic psychosocial stress and the primary outcome measure for this study. The PSS-10 is one of the most widely used psychosocial stress scales and assesses the extent to which an individual appraises their life as unpredictable, uncontrollable, or overloaded over the past month (scale: 0 = never, 4 = very often). Higher total scores on this 10-item measure reflect greater chronic psychosocial stress. The PSS-10 demonstrated good reliability in this sample at pre-test (T0) and post-test (T1) (*α*_*T*0_ =.93; *α*_*T*1_ =.80).

To provide converging evidence that changes in this scale were reliable, we additionally assessed several secondary outcomes that should covary with PSS-10 scores. This included depression, anxiety, and stress symptoms using subscales of the Depression Anxiety Stress Scale (DASS-21) [36], and subjective wellbeing using the World Health Organization Wellbeing Index (WHO-5) [37]. Higher total scores on the DASS-21 represent poorer mental health outcomes, while higher total scores on the WHO-5 represent greater wellbeing. Each secondary outcome measure demonstrated adequate reliability at each time point (DASS-21: depression *α*_*T*0_ =.87, *α*_*T*1_ =.79; anxiety *α*_*T*0_ =.64, *α*_*T*1_ =.81; stress *α*_*T*0_ =.64, *α*_*T*1_ =.73; WHO-5: wellbeing *α*_*T*0_ =.86, *α*_*T*1_ =.83).

#### Dispositional mindfulness

The Mindful Attention Awareness Scale (MAAS) [38] was used to assess dispositional use of mindfulness. The MAAS is a widely used 15-item scale that examines how frequently individuals experience an absence of attention to and awareness of present moment experiences (scale: 1 = almost always, 6 = almost never). Higher mean scores indicate higher dispositional mindfulness. The MAAS demonstrated good reliability in this sample (*α*_*T*0_ =.86; *α*_*T*1_ =.85).

#### Cognitive reappraisal

The cognitive reappraisal subscale of the Emotion Regulation Questionnaire (ERQ-CR) [39] was used to assess dispositional use of cognitive reappraisal. This 6-item scale assesses the extent to which an individual uses cognitive reframing of situations to change how they feel about an emotional experience (scale: 1 = strongly disagree, 7 = strongly agree). Higher mean scores indicate higher dispositional use of cognitive reappraisal. The ERQ-CR had good reliability (*α*_*T*0_ =.91; *α*_*T*1_ =.93).

#### Repetitive negative thinking

Maladaptive rumination (past-oriented) and worry (future-oriented) were used to assess the disposition to engage in self-focused, repetitive, and perseverative thought processes that maintain negative affect during times of psychosocial stress (i.e., maladaptive emotion regulation strategies).

The maladaptive form of rumination was examined using the 5-item brooding subscale of the Ruminative Responses Scale (RRS-BR) [40], where higher scores indicate greater use of negative self-reflection and a focus on obstacles to problems when experiencing a negative mood (scale: 1 = almost never, 4 = almost always). The reflection subscale of this scale was excluded because it captures positive aspects of rumination and has been shown to suffer from a poor factor structure [41]. The RRS-BR had good reliability (*α*_*T*0_ =.88; *α*_*T*1_ =.86). Worry was examined using the 16-item Penn State Worry Questionnaire (PSWQ) [42]. The PSWQ assesses the generality, excessiveness, and uncontrollability and an individuals’ disposition to worry (scale: 1 = not at all typical of me, 5 = very typical of me). Higher total scores indicate a higher disposition to worry. The PSWQ had good reliability in this sample (*α*_*T*0_ =.96; *α*_*T*1_ =.91).

#### Cardiorespiratory fitness

Maximal aerobic capacity $\left (\dot {V}O_{2max}\right)$ and aerobic economy were estimated using a graded incremental exercise protocol on a motorized treadmill [43]. During the exercise protocol, a 1% treadmill gradient was maintained to match the energy-cost of outdoor running [44]. Prior to beginning the protocol, participants completed a 2–3-min warm-up of treadmill running at a low-intensity velocity of their choosing. The protocol began with treadmill running at 6 km/h, and velocity (i.e., exercise intensity) was increased in increments of 2 km/h every 3 min until volitional exhaustion or until $\dot {V}O_{2max}$ criteria were obtained.

The final minute of treadmill running at each velocity (i.e., 2 to 3 min) represented the steady-state for a given exercise intensity (e.g., 6, 8, 10, 12, and 14 km/h). At each steady-state period, heart rate was recorded using a Polar heart rate monitor (Polar Electro, Kempele, Finland) and perceived exertion was recorded using the Borg Rating of Perceived Exertion (RPE) scale [45]. Oxygen consumption ($\dot {V}O_{2}$ L/min) was measured continuously using open-circuit spirometry (Vmax Encore Metabolic Cart, Carefusion, San Diego, CA, USA). Criteria for $\dot {V}O_{2max}$ were volitional exhaustion, or when two of the following were satisfied: (1) a plateau in oxygen consumption despite increasing work rate, (2) a heart rate more than 90% predicted (220 beats/min minus age in years), or (3) a respiratory exchange ratio greater than 1.15.

Four measures of aerobic economy were obtained in the final minute of each running velocity (i.e., each steady-state exercise intensity). This included (1) absolute oxygen cost ($\dot {V}O_{2}$ L/min), (2) relative oxygen cost (i.e., oxygen cost relative to $\dot {V}O_{2max}$; $\% \dot {V}O_{2max}$), (3) heart rate (beats/min), and perceived exertion (RPE). The percentage of participants reaching each intensity were as follows: 6 km/h = 100%, 8 km/h = 100%, 10 km/h = 82.4%, 12 km/h = 76.5%, and 14 km/h = 23.5%. Therefore, to assess within-group changes in aerobic economy with respect to time and across the broadest range of exercise intensities, analyses of aerobic economy were restricted to the 76.5% of participants with full data across 6 to 12 km/h (i.e., *n* = 13).

To ensure test-retest reliability, testing protocols at T0 and T1 were conducted under identical conditions (temperature, 20–25 ^∘^C; humidity, ≤ 60%; air circulation controlled at 10 km/h using a motorized fan; assessments at T0 and T1 were completed at the same time within ± 2 h). Participants were prohibited exercise, alcohol, or caffeine consumption within 24 h of testing, and diet was restricted (i.e., mandatory consumption of a small meal containing carbohydrates 2 h prior to exercise and no further food until test completion).

## Statistical analysis

All data were analyzed using R statistical software (v. 3.5.1; R Core Team) on R Studio (v. 1.1.456; RStudio, Inc) for Windows 10.

### Sample size planning for a future definitive trial

First, the mean gain score for changes in chronic psychosocial stress (PSS-10) was divided by the standard deviation of the gain scores, yielding the operative effect size *d*_*z*_. Second, a safeguard effect size was computed by calculating the lower limit of a one-sided 80% CI on *d*_*z*_ (conf.limits.nct: MBESS package v-4.6.0) [46]. A safeguard effect size is designed to guard against imprecise estimation of pilot effect estimates by decreasing the magnitude of an effect based on the width of its confidence interval [47]. These two effect size estimates (hereby population estimates: *δ*_*z*_) were then used as input to an accuracy in parameter estimation sample size analysis with 99% assurance (ss.aipe.sm: MBESS package). This process was repeated for hypothetical operative between-group *d* statistics (hereby: *δ*_*s*_) ranging from 0.8 to 0.2 (ss.aipe.smd: MBESS package). The latter analysis estimated the sample size required to detect hypothetical differences (with precision) between T1–T0 gain scores of different arms of a confirmatory RCT.

### Estimation of participant-centered outcomes

It is important to note that the inferential statistics reported alongside estimations of trial treatment effects should be considered exploratory. These findings are not meant to represent the results of a future definitive trial.

#### Psychosocial stress factors, mindfulness, emotion regulation, and $\dot {V}O_{2max}$

Responses to these variables were analyzed using one-sample *t* tests on T1–T0 gain scores (t.test: base R) (sampling units: *n* = 17; observations = 34). As a sensitivity test to any assumption violations, these variables were also analyzed using robust methods based on T1–T0 contrasts of the 20% trimmed mean difference using a percentile bootstrap approach (trimpb: Rallfun package v-35; bootstrap samples = 2000) [48]. Unstandardized effect sizes were reported as the T1–T0 gain score with 95% CIs. Standardized effect sizes were reported as Cohen’s *d* using the standard deviation of the pre-test scores as standardizer (hereby *d*_*pretest*_), as recommended in Glass et al. [49] for pre-post designs. This corresponded to the equation:

1 $$  d_{pretest} = \frac{M_{T1} - M_{T0}}{SD_{T0}}  $$

In the above equation, *M*_*T*1_ refers to the mean of the post-test, *M*_*T*_ refers to the mean of the pre-test, and *SD*_*T*0_ refers to the standard deviation of the pre-test. The 95% CIs on *d*_*pretest*_ were computed using the *bias-corrected-and-accelerated* (BCa) bootstrap method with 2000 resamples (boot: boot package v-1.3-22) [50]. Interpretation of *d*_*pretest*_ is consistent with Cohen’s *d* for between-group designs.

#### Aerobic economy

These variables included absolute oxygen cost, relative oxygen cost, heart rate, and perceived exertion (RPE) (sampling units: *n* = 13; observations = 104). These data were arrayed as separate 2 (time: T1 vs. T0) by 4 (velocity: 6, 8, 10, and 12 km/h) within-within factorials and analyzed using linear mixed models fit by REML (lmer: lmerTest package v-3.1-0) [51]. Fixed effect intercepts were included for time, velocity, and their interaction, and random effect intercepts were included for participants. The final model equation was:

2 $$ y \sim \text{time} \ \times \ \text{velocity} + (1 \ | \ \text{participant})  $$

To determine whether random participant intercepts improved model fit, REML-likelihood ratio tests of the model with and without random intercepts were performed (ranova: lmerTest package). Satterthwaite’s degrees of freedom was used to evaluate the significance of *F* tests for fixed effects terms (anova: lmerTest package) and *t* tests for follow-up pairwise comparisons (emmeans: emmeans package v-1.3.2) [52], as recommended by [53]. Fixed effects yielding *p* <.05 were followed up with pairwise contrasts of T1–T0 gain scores. Unstandardized effect sizes were reported as T1–T0 gain scores with 95% CIs. Because of the method by which variance is partitioned in mixed models [54], standardized effect sizes were not computed as there is no widely agreed-upon method of doing so.

## Results

**Fig. 1:**
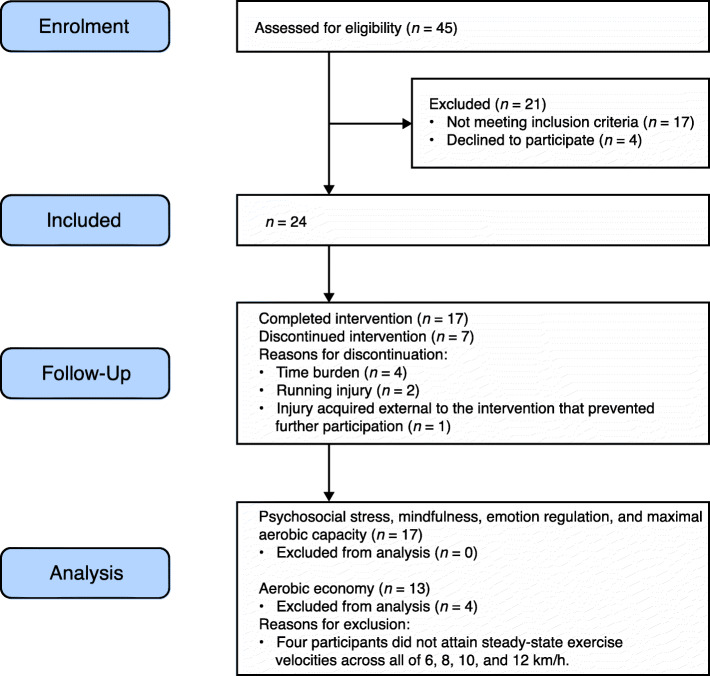
CONSORT flow chart

Figure 1 shows the Consolidated Standards of Reporting Trials (CONSORT) diagram for participant flow through the study. Twenty-four participants were allocated to the intervention and 70.8% provided full data. The final sample size was *N* = 17 (baseline data is given in Table 1). This satisfied our sample requirements, and no attempts were made toward further data collection. All participants who completed this study met the adherence requirements, and the overall dosage results are reported in Table 2.

**Table 1:** Baseline demographic characteristics of participants

	All participants	PSS declined	PSS increased or no change
Total *n* (%)	17 (100.00%)	12 (70.59%)	5 (29.41%)
Mean baseline PSS-10 (*SD*)	20.12 (7.56)	23.50 (5.35)	12.00 (5.74)
Mean age in years (*SD*)	22.88 (2.71)	23.08 (2.84)	22.40 (2.61)
Gender: male *n* (%)	8 (47.06%)	5 (29.41%)	3 (17.65%)
Gender: female *n* (%)	9 (52.94%)	7 (41.18%)	2 (11.76%)
Ethnicity: Caucasian *n* (%)	11 (64.71%)	7 (41.18%)	4 (23.53%)
Ethnicity: Asian *n* (%)	6 (35.29%)	5 (29.41%)	1 (5.88%)
Mean baseline $\dot {V}O_{2max}$	45.34 (7.44)	45.81 (5.58)	44.20 (11.54)
Mean baseline BMI (*SD*)	23.29 (2.65)	23.96 (2.45)	21.68 (2.64)

*SD* = standard deviation; *n* = count; *PSS-10* = Perceived Stress Scale; $\dot {V}O_{2max}$ = maximal aerobic capacity; *BMI* = body mass index

**Table 2:** Summarized dosage results

	*M* (*SD*)	Range
MBI attendance (%)	79.41 (11.64)	62.50–100.00
Formal meditation (h)	24.81 (2.69)	19.33–29.42
Total running sessions	34.76 (5.39)	25.00–42.00
Mean exercise intensity (% $\dot {V}O_{2max}$)	70.54 (6.69)	56.78–82.15
Running distance (km)	228.10 (81.67)	143.80–474.27
Running time (h)	24.50 (6.82)	17.99–45.37

*M* = mean; *SD* = standard deviation; *h* = hours; *km* = kilometers; $\dot {V}O_{2max}$ = maximal aerobic capacity; *MBI* = mindfulness-based intervention

### Protocol feasibility

#### Retention rate

Of the 24 participants allocated into the study, *n* = 17 completed the intervention (retention rate = 70.83%).

#### Assessment response rate

All participants provided paired data points for chronic psychosocial stress, mindfulness, use of emotion regulation strategies, and $\dot {V}O_{2max}$ (response rate = 100%). For assessments of aerobic economy, *n* = 17 participants reached a steady-state velocity at 6 and 8 km/h (100%), *n* = 14 reached 10 km/h (82.4%), *n* = 13 reached 12 km/h (76.5%), and *n* = 4 reached 14 km/h (23.5%).

#### Recruitment rate and sample size planning for a future definitive trial

Of the 45 participants who expressed interest in participation, *n* = 24 met the inclusion criteria and agreed to participate (recruitment rate = 53.3%). The results of the sample size planning analysis are presented in Table 3. It is feasible to detect within-group changes (with precision) up to *δ*_*z*_ = −0.37 and between-group differences in gain scores (with precision) up to *δ*_*s*_ = 0.50. Between-group differences in gain scores of *δ*_*s*_ = 0.40 are detectable with 80% power, but without precision. Detection of small between-group effects below these values is infeasible.

**Table 3:** Range of sample sizes required to estimate PSS-10 effects for a confirmatory RCT comparing aerobic exercise, mindfulness, combination training, and a control arm

Population effect	Target *MoE*	*N* required per cell	*N* adjusted	*N* required per cell per trial	Feasible
*δ*_*z*_					
−0.60	0.30	63	89	9	Yes
−0.37	0.18	132	186	19	Yes
*δ*_*s*_					
0.80	0.40	59	84	9	Yes
0.70	0.35	73	103	11	Yes
0.60	0.30	96	136	14	Yes
0.50	0.25	133	188	19	Yes
0.40	0.20	202	285	29	*Yes
0.30	0.15	352	496	50	No
0.20	0.10	779	1098	110	No

*δ*_*z*_ = within-group (T1–T0) gain in PSS-10; *δ*_*s*_ = between-group differences in gain scores between different arms of a future RCT; *MoE* = margin-of-error (i.e., the 95% confidence interval half-width); *N* = number of participant; *per cell* = paired observations (*δ*_*z*_) or observations per arm of a future RCT (*δ*_*s*_), *Nadjusted* = *N* required per cell adjusted for the retention rate observed in the pilot study. Feasibility is judged against 10 repetitions of the trial over a 5-year period with a maximum of *N* = 20 per treatment arm across each repetition (i.e., total *N* = 80 per trial). Yes = trial is feasible. *Yes = trial is not feasible using accuracy in parameter estimation criteria; however, the effect is detectable with 80% power. No = trial is infeasible

### Estimation of participant-centered outcomes

#### Psychosocial stress factors, mindfulness, emotion regulation, and $\dot {V}O_{2max}$

Summarized results of these analyses are given in Table 4.

**Table 4:** Summarized results for pre-test (T0), post-test (T1), and gains (T1–T0) for psychosocial stress factors, mindfulness, emotion regulation factors, and maximal aerobic capacity

	T0: *M* (*SD*)	T1: *M* (*SD*)	Gain: *M* (*SD*) [95% CI]	*d*_*pretest*_ [95% CI]	*t*	*p*	*p*_*R*_
**Psychosocial stress**							
PSS-10	20.12 (7.56)	15.88 (4.54)	−4.24 (7.01) [ −7.84,−0.63]	−0.56[ −1.14,−0.06]	−2.49	.024	.020
DASS-21 (S)	28.59 (6.07)	24.00 (5.87)	−4.59 (4.17) [ −6.73,−2.44]	−0.76[ −1.24,−0.52]	−4.54	<.001	<.001
DASS-21 (A)	19.88 (4.82)	20.00 (6.60)	0.12 (4.55) [ −2.22,2.46]	0.02 [ −0.43,0.53]	0.11	.916	.789
DASS-21 (D)	23.76 (7.21)	19.76 (4.63)	−4.00 (6.32) [ −7.25,−0.75]	−0.56[ −0.98,−0.15]	−2.61	.019	.015
WHO-5	54.35 (18.55)	64.00 (12.65)	9.65 (15.75) [1.55, 17.75]	0.52 [0.19, 1.02]	2.53	.023	.028
**Mindfulness**							
MAAS	3.78 (0.70)	4.00 (0.66)	0.22 (0.75) [ −0.17,0.60]	0.31 [ −0.24,0.88]	1.18	.256	.225
**Emotion regulation**							
ERQ-CR	4.31 (1.46)	5.18 (1.18)	0.86 (1.04) [0.33, 1.39]	0.59 [0.22, 1.15]	3.43	.003	.003
RRS-BR	11.00 (4.14)	9.59 (3.36)	−1.41 (2.55) [ −2.72,−0.10]	−0.34[ −0.75,−0.07]	−2.28	.037	.051
PSWQ	50.12 (16.19)	43.94 (11.26)	−6.18 (10.05) [ −11.34,−1.01]	−0.38[ −0.78,−0.05]	−2.53	.022	.012
**Aerobic capacity**							
$\dot {V}O_{2max}$	45.34 (7.44)	45.58 (8.91)	0.25 (5.98) [ −2.83,3.32]	0.03 [ −0.51,0.42]	0.17	.868	.642

Sampling units: *N* = 17 and observations = 34. Degrees of freedom for Student’s *t* test = 16. *M* mean, *SD* = standard deviation; *CI* = confidence interval; *d*_*pretest*_ = Cohen’s *d* with pre-test standard deviation as standardizer; *p* = *p* value; *p*_*R*_ = robust *p* value; *PSS* = Perceived Stress Scale; *DASS-21* = Depression Anxiety Stress Scales (*S* = stress; *A* = anxiety; *D* = depression), *WHO-5* = World Health Organization Wellbeing Index; *MAAS* = Mindful Attention Awareness Scale; *ERQ-CR* = cognitive reappraisal subscale of the Emotion Regulation Questionnaire; *RRS-BR* = brooding subscale of the Ruminative Responses Scale; *PSWQ* = Penn State Worry Questionnaire; $\dot {V}O_{2max}$ = maximal aerobic capacity

##### Chronic psychosocial stress

**Fig. 2:**
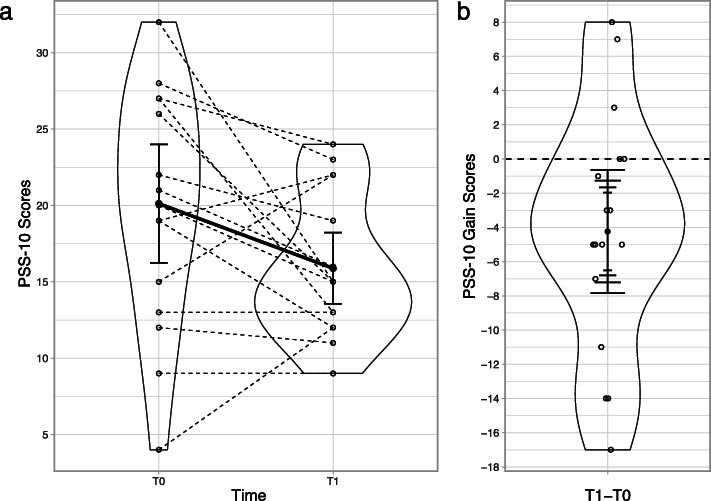
**a** Means and 95% confidence intervals (CIs) at pre-test (T0) and post-test (T1) for responses to the PSS-10. Unfilled circles represent individual participant data points, and dashed lines join paired responses for each participant. **b** T1–T0 gain scores for the PSS-10, where (from largest to smallest) the error bar represents 95%, 90%, 85%, and 80% CIs. Unfilled circles represent individual gain scores, and a dashed line marks the null hypothesis of zero gain. PSS-10 = Perceived Stress Scale

There was an overall reduction in our primary dependent measure of chronic psychosocial stress (PSS-10; *M* = −4.24, 95% CI [ −7.84,−0.63], *p* =.024; see Fig. 2). This effect was also robust (*p*_*R*_ =.020). Twelve participants reported a reduction on the PSS-10, and five participants reported no change or an increase. In common language, there was a 72.7% chance that a participant picked at random would report a lower PSS-10 score following the study compared to before the study. Because seven participants dropped out of the study, we also assessed whether survivorship bias was a threat to the validity of this test result. To this end, we conducted a *last observation carried forward* analysis which included the last known state of the subject in the analysis under the assumption they would report no change had they completed the study. This analysis yielded a small decline in effect size magnitude, but the test remained statistically significant (*M* = −3.00 95% CI [ −5.61,−0.39], *p* =.026, *p*_*R*_ =.018).

Reductions in PSS-10 were corroborated by improvement in several secondary stress measures. There was an overall reduction in stress symptoms (*M* = −4.59, 95% CI [ −6.73,−2.44], *p* <.001) and depressive symptoms (*M* = −4.00, 95% CI [ −7.25,−0.75], *p* =.019), and an overall improvement in global wellbeing (*M* = 9.65, 95% CI [1.55, 17.75], *p* =.023). These effects were also robust. However, we are insufficiently confident to comment on the direction of change in anxiety (*M* = 0.12, 95% CI [ −2.22,2.46], *p* =.916). A robust test did not change this conclusion.

##### Mindfulness

Participants reported an overall increase in dispositional mindfulness (*M* = 0.22, 95% CI [ −0.17,0.60], *p* =.256). However, these data are compatible with a 0.17 unit decrease in mindfulness as well as a 0.60 unit increase in mindfulness. Therefore, we are insufficiently confident to comment on the direction of this change. A robust test did not change this conclusion.

##### Emotion regulation

There was a mean improvement in the use of cognitive reappraisal (*M* = 0.86, 95% CI [0.33, 1.39], *p* =.003). This effect was robust. There was a mean reduction in the use of maladaptive rumination (*M* = −1.41, 95% CI [ −2.72,−0.10], *p* =.037). However, this effect was not robust (*p*_*R*_ =.051). Finally, there was an overall reduction in the disposition to worry (*M* = −6.18, 95% CI [ −11.34,−1.01], *p* =.022). This effect was also robust.

##### $\dot {V}O_{2max}$

There was a negligible change in $\dot {V}O_{2max}$ (*M* = 0.25, 95% CI [ −2.83,3.32], *p* =.868). However, we are insufficiently confident to comment on the overall direction of this change. A robust test did not change this conclusion.

#### Aerobic economy

Summarized results of aerobic economy analyses are given in Table 5. Modeling variability in participant intercepts improved model fit for each mixed model (*p*s <.001; see Supplementary Results Tables 1 through 4). The assumptions of linearity, homoskedasticity, and normality of residuals were adequate for each mixed model, and a sensitivity test revealed no evidence of participant and response-level outliers in each model.

**Table 5:** Summarized Type III ANOVA table of the linear mixed model (fit by REML) for each aerobic economy outcome

	*SS*	*MS*	*df*	*F*	*p*
**Oxygen Cost**					
Main Effect of Time	0.49	0.49	1,84	7.57	.007
Main Effect of Velocity	40.23	13.41	3,84	209.18	<.001
Time × Velocity Interaction	0.23	0.08	3,84	1.20	.316
**Relative Oxygen Cost**					
Main Effect of Time	495.99	495.99	1,84	16.09	<.001
Main Effect of Velocity	35315.81	11771.94	3,84	381.90	<.001
Time × Velocity Interaction	175.98	58.66	3,84	1.90	.135
**Heart rate**					
Main Effect of Time	541.73	541.73	1,84	8.13	.005
Main Effect of Velocity	47653.51	15884.50	3,84	238.43	<.001
Time × Velocity Interaction	51.82	17.27	3,84	0.26	.855
**Perceived Exertion (RPE)**					
Main Effect of Time	13.16	13.16	1,84	13.93	<.001
Main Effect of Velocity	673.57	224.52	3,84	237.52	<.001
Time × Velocity Interaction	1.49	0.50	3,84	0.53	.666

*Note.* Sampling Units: *N* = 13 and observations = 104; SS = sum of squares; MS = mean square; *F* = *F* statistic; *df* = degrees of freedom; *p* = *p* value. *F* tests use Satterthwaite’s degrees of freedom

##### Absolute oxygen cost

**Fig. 3:**
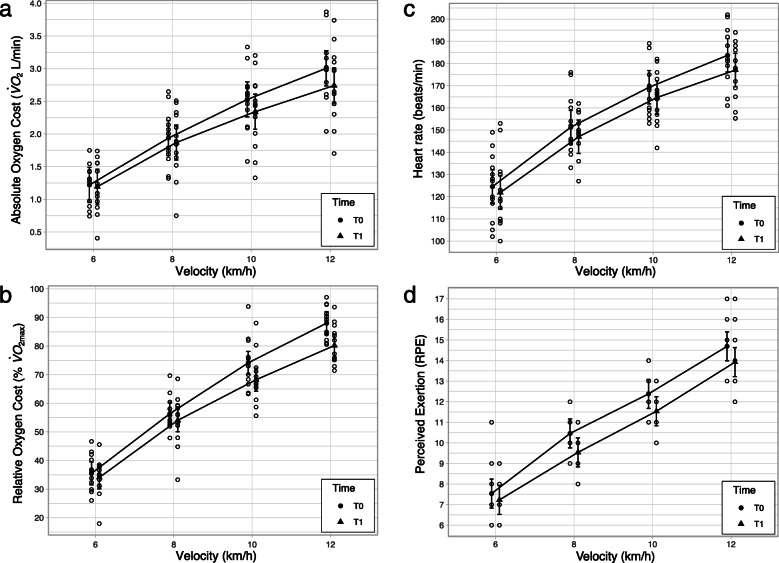
Dot plots of aerobic economy data across velocity and time for **a** absolute oxygen cost ($\dot {V}O_{2max}$ L/min), **b** relative oxygen cost ($\% \dot {V}O_{2max}$), **c** heart rate (beats/min), and **d** perceived exertion (RPE). The estimated marginal means for each velocity at pre-test (T0) are marked by filled black circles, and at post-test (T1) are marked by filled black triangles. Each unfilled circle represents an individual participant data point. Error bars represent 95% CIs

There was a statistically significant main effect of time (*F*(1, 84) = 7.57, *p* =.007) and velocity (*F*(3, 84) = 209.18, *p* <.001). We are insufficiently confident to comment on the presence of an interaction (*F*(3, 84) = 1.20, *p* =.316). A pairwise (T1–T0) contrast of the main effect of time indicated that, on average, there was a change in oxygen cost across all velocities of $-0.14 \dot {V}O_{2}$ L/min (95% CI [ −0.24,−0.04]; see Fig. 3a).

##### Relative oxygen cost

There was a statistically significant main effect of time (*F*(1, 84) = 16.09, *p* <.001) and velocity (*F*(3, 84) = 381.90, *p* <.001). We are insufficiently confident to comment on the presence of an interaction at a.05 threshold (*F*(3, 84) = 1.90, *p* =.135). A pairwise (T1–T0) contrast of the main effect of time indicated that, on average, there was a decrease in $\%\dot {V}O_{2max}$ across all velocities following the study (*M*= −4.37, 95% CI [ −6.53,−2.20]; see Fig. 3b).

##### Heart rate

There was a statistically significant main effect of time (*F*(1, 84) = 8.13, *p* =.005) and velocity (*F*(3, 84) = 238.43, *p* <.001). We are insufficiently confident to comment on the presence of an interaction (*F*(3, 84) = 0.26, *p* =.855). A pairwise (T1–T0) contrast of the main effect of time indicated there was, on average, a decrease in heart rate across all velocities (*M* = −4.56, 95% CI [ −7.75,−1.38]; see Fig. 3c).

##### Perceived exertion (RPE)

There was a statistically significant main effect of time (*F*(1, 84) = 13.93, *p* <.001) and velocity (*F*(3, 84) = 237.52, *p* <.001). We are insufficiently confident to comment on the presence of an interaction (*F*(3, 84) = 0.53, *p* =.666). A pairwise (T1–T0) contrast of the main effect of time indicated there was, on average, a decrease in RPE across all velocities following the study (*M* = −0.71, 95% CI [ −1.09,−0.33]; see Fig. 3d).

## Discussion

### Protocol feasibility

This study was a single-arm pre-post pilot and feasibility study of an intervention comprised of aerobic exercise and mindfulness-based training delivered concurrently over 16 weeks. The first aim of this study was to obtain sufficient assurance of protocol feasibility to determine if a definitive RCT comparing aerobic exercise, mindfulness, combination training, and a control arm was an appropriate trial design. We considered retention rate, assessment response rate, recruitment rate, and required sample size for a confirmatory trial as measures of feasibility. The retention rate was 70.8%, which was above the required rate of 65.0%. This is also above the lower limit of retention identified for mindfulness-based intervention (MBI) training in healthy individuals [6] and for previously non-exercising individuals [35]. This indicates that the current intervention may be feasible when compared to already existing interventions that yield salutary mental health outcomes. Furthermore, the assessment response rate for pre-post measures with single paired data points was 100%. This included our primary dependent outcome measure of chronic psychosocial stress (PSS-10). This indicates that the trial is feasible from a data collection perspective. However, participants varied in the steady-state velocity attained during assessments of aerobic economy. This was unavoidable given that participants differed in cardiorespiratory fitness at baseline. For this reason, it may be necessary to continue to regard aerobic economy as a secondary measure based on a subgroup analysis in a future confirmatory trial. Finally, given that the combination treatment arm comprises all elements of a future RCT and is the most labor intensive for participants, these rates may also be reasonable estimates for each arm of a future RCT.

The study recruitment rate was 53.3%. Across a 2-month recruitment period (i.e., 1 month of recruitment at each wave of a future definitive trial) and with a total of 45 applicants, this is indicative of a recruitment potential of 12 participants per month for the combination trial arm of a future RCT. With an expanded research team for recruitment, assessment, and facilitation of the intervention, a recruitment potential of 12 participants per month *per treatment arm* of an RCT (i.e., four arms: *n* = 48) may represent a reasonable estimate of recruitment feasibility. Based on our sample size analysis, precise estimation of within-group changes in PSS-10 gain scores requires between 89 (*δ*_*z*_ = −0.60) and 186 (*δ*_*z*_ = −0.37) paired observations. A precise estimate of differences in gain scores between each arm of an RCT will require between 84 (large difference: *δ*_*s*_ = 0.80) and 1098 (small difference: *δ*_*s*_ = 0.20) observations per cell. These calculations reflect a retention rate of 70.8%.

Under the assumption that an RCT is only feasible with *n* = 20 per treatment arm (i.e., total *N* = 80 per trial) across 10 trial repetitions over 5 years, the total maximum number of participants is *n* = 200 per treatment arm (i.e., total *N* = 800). A recruitment rate of *n* = 12 per treatment arm per month is therefore feasible to fill each trial run following the second month of the protocol (estimated sample at month 2: *n* = 24 per treatment arm) with a start date at month 3 following assessments. Assuming recruitment continues ongoing each month, each trial start date will satisfactorily attain *n* = 20 participants per treatment arm. Assuming a retention rate of 70.8%, a total recruitment of *n* = 200 per treatment arm by the end of the protocol is satisfactory to detect within-group changes in PSS-10 gain scores (with precision) as low as *δ*_*z*_ = −0.37, and between-group difference in gain scores (with precision) up to *δ*_*s*_ = 0.50. Differences in gain scores of *δ*_*s*_ = 0.40 will be detectable with 80% power, but these effects may have lower than desired precision. Estimation of very small differences in gain scores (i.e., *δ*_*s*_≤ 0.30) is infeasible. Based on these criteria, we conclude that there is sufficient assurance of protocol feasibility to conduct a confirmatory RCT under the above assumptions and detect most effects precisely (or as a minimal requirement, statistically).

Of course, however, these above assumptions do not take into consideration additional uncertainties that may impact the capacity of a single research group to conduct a confirmatory trial. This includes uncertainty in future economic conditions, finances, and resource availability. Therefore, this confirmatory trial may be more appropriately suited to a large multisite clinical trial that spreads risk of conducting 10 trial repetitions across multiple research groups. This approach has the additional benefit of evaluating the trial treatment effect across large and heterogeneous populations in varied treatment settings, and will therefore mitigate multiple threats to the validity of observed effects.

Additional considerations for a future definitive trial may also include ensuring that the effects of the intervention are not driven by differences in baseline stress levels or baseline cardiorespiratory fitness levels that differ across groups. While randomization ensures that these differences, on average, should be equal across treatment arms, one option would be to perform block stratified random assignment on factors that might bias results for each trial repetition. With regard to baseline stress, this could include block randomization based on a simple one-item question about overall stress levels that is embedded in the initial application to the intervention, as recommended by Shadish and colleagues [55]. This is particularly important for extending the program to the general public, which, unlike our student sample, does not necessarily have specific low-stress periods within the calendar year to serve as baseline. With respect to cardiorespiratory fitness, baseline levels of $\dot {V}O_{2max}$ might also be important to consider within the block stratified random assignment procedure, particularly if the effects of the intervention are driven, in part, by changes in cardiorespiratory fitness.

A further consideration for a future definitive trial may be to broaden the trial eligibility criteria to allow findings to be more generalizable. Currently, the eligibility criteria only allowed for young and relatively healthy adults as part of a prevention-focused intervention. While this population was the target of interest, it may not be the population that needs this intervention most. However, any deviation from the methods and protocol of this study may require further pilot testing prior to performing a definitive trial.

### Estimation of participant-centered outcomes

The second aim of this study was to estimate the range and direction of within-group changes in chronic psychosocial stress (PSS-10) in a nonclinical sample. Our analysis indicated that, overall, chronic psychosocial stress (as measured by the PSS-10 scale) declined following the study. Reductions on the PSS-10 scale of the magnitude observed in this study have been considered clinically meaningful in past research utilizing this measure [56–58]. The meta-analytic estimate of the effect of (controlled) mindfulness-based interventions on stress outcomes in healthy participants has been reported as *d* = −0.74 [ −1.07,−0.41] [6]. If this effect estimate and confidence interval represents the average effect of mindfulness-based training on stress-related measures, our current observed effect size of −0.56 could be considered average or moderate in magnitude. This standardized change is also within the range reported meta-analytically for mental health improvements observed following aerobic exercise interventions [5, 20]. Based on these observations, there is insufficient evidence to suggest that combination training yields improvements in chronic psychosocial stress over and above each intervention alone. However, the confidence interval (CI) on our effect indicates that the estimate of change in PSS-10 in this study is very imprecise. The 95% CIs on this effect are consistent with reductions in PSS-10 that could be considered smaller than average, or even above average.

Further support for the validity of a reduction in PSS-10 scores comes from changes in our secondary mental health outcome measures. This included a reduction in stress and depressive symptoms, and an improvement in global wellbeing. Indeed, mean improvement in wellbeing was of a magnitude of approximately 10 percentage points on the WHO-5 scale, which is indicative of a clinically relevant improvement with respect to the scale [59]. However, we are insufficiently confident to comment on the direction and magnitude of change in anxiety symptoms.

Participants reported an overall increase in dispositional mindfulness. This represented a partial unit change from *somewhat frequently* to *somewhat infrequently*, in response how frequent participants reported an absence of attention to or awareness of present moment experiences. However, the confidence intervals on these data are also compatible with a partial unit decrease in mindfulness, as well as a greater than half unit increase in mindfulness. Therefore, we are insufficiently confident to comment on the direction of change of this effect in the present study.

Dispositional mindfulness represents the quality of attention and awareness with which one attends to the present moment experience, and is considered a core mechanistic component of how MBIs exert their effects [10, 60]. In the present study, participants reported an average of 24.81 h of formal meditation practice, which is within the range of practice reported meta-analytically in a typical MBI in healthy participants [6]. The meta-analytic estimate of the effect of MBIs on mindfulness in the above publication was *d* = 0.60, 95% CI [0.36, 0.85]. The small magnitude of change in mindfulness in the present study is therefore unexpected (i.e., *d*_*pretest*_ = 0.31, 95% CI [ −0.24,0.88]). However, it must be noted that measures of mindfulness are not consistently reported for MBI interventions [6], and over half of the studies reported in a recent meta-analysis found no significant effect following MBI training [61].

Participants reported an overall increase in use cognitive reappraisal. In descriptive terms, this represented a unit change from *neither agree or disagree* to *agree*, in response to questions evaluating the disposition to use this emotion regulation strategy. Evidence from intervention studies [62, 63] demonstrates that mindfulness and MBI training are often implicated with a greater capacity for this emotion regulation strategy. Improvement in cognitive reappraisal is also considered central to some mindfulness theories [62], although it is considered non-essential or secondary to others [18]. Beyond reappraisal, participants reported a decrease in the use of maladaptive rumination and worry. Compared to the meta-analytic estimate of the effect of mindfulness-based training on stress [6], these effects might be considered small in magnitude. A reduction in these aspects of repetitive negative thinking has been implicated in meta-analyses as mechanisms of mindfulness [15]. However, the effect of this study on rumination was not robust. That is, when sampling from a population where a null hypothesis of no change in rumination in the majority of individuals is true, the magnitude of change in this parameter in the present study is expected to occur at a rate of *p*_*R*_ =.051 (i.e., 5.10% of the time). Notably, there were no outliers in this variable and no evidence of a strong deviation from normality, meaning that the standard (i.e., non-robust) test may be a more appropriate reflection of the data. This interpretation is strengthened by observation that Alderman and colleagues [7] reported a statistical reduction in rumination following combination training in their study. Although, we must caution over-interpretation of these small sample pilot results given their large CIs.

Finally, with respect to cardiorespiratory fitness, there was a negligible overall change in $\dot {V}O_{2max}$ following the intervention. On average, participants reported a $\dot {V}O_{2max}$ value between 45 and 46 at both time points. Meta-analytic evidence suggests that aerobic interventions similar to the so-called Hickson protocol [64] are most reliably implicated in improvement in this parameter [65]. This involves 10 weeks of aerobic exercise involving a combination of endurance training (i.e., consistent exercise for a specified time period without break) and interval training (i.e., bursts of high-intensity output ≥ 91% $\dot {V}O_{2max}$). While the current intervention involved a combination of continuous and interval training, it is plausible the mean intensity at which participants conducted exercise (i.e., 70% of their $\dot {V}O_{2max}$) and frequency (i.e., 2.32 runs per week) was insufficient to yield improvement in overall $\dot {V}O_{2max}$. Given that the present trial intervention may be unlikely to improve $\dot {V}O_{2max}$, one option for a future trial would be to include additional measures of cardiorespiratory fitness. This might include assessment of specific fitness objectives (e.g., a 500 meter run) as secondary fitness outcomes.

However, there were improvements in aerobic economy across steady-state (submaximal) exercise velocities in our subgroup analyses. This included a reduction in absolute oxygen cost, relative oxygen cost, heart rate, and perceived exertion. Improvement in indices of aerobic economy is indicative of a cascade of metabolic and cardiopulmonary effects that result in better use of oxygen (i.e., increased energy production) relative to a given exercise intensity. This includes more efficient cardiorespiratory responses (e.g., lower heart rate), thermoregulation (e.g., changes in core body temperature), and substrate metabolism [26]. The relationship between these parameters and mental health is relatively understudied. However, several theories assert that exercise adaptations such as these may be associated with resilience to acute psychosocial stress, with potential implications for overall mental health [23]. It is therefore plausible that the salutary effects following training in this intervention are partially determined by improvements in aerobic economy. However, note well that these effects are imprecise. This is reflected in the large CIs on each of the reported effects.

### Strengths, limitations, and generalizability

Notable strengths of this study included the following: (1) use of a full mindfulness psychoeducation program in contrast to a single mindfulness component (e.g., focused-attention meditation alone); (2) use of an aerobic exercise program that prescribed exercise of a duration, frequency, and intensity that has been clinically shown to improve or maintain aerobic fitness; (3) complete individualization of aerobic exercise prescriptions through objective cardiorespiratory fitness assessments; (4) comprehensive monitoring of training compliance through GPS data; (5) provision of a detailed methodological protocol that is presented here in sufficient detail to allow for replication; and (6) extensive quantification of multiple protocol feasibility criteria.

However, the clearest limitation is that a single-arm study design presents with difficulty in distinguishing between the effects of the treatment and several threats to validity. For example, the observed reduction in chronic psychosocial stress may be an effect of intervention exposure, but it may also be partly explained through a placebo effect, maturation, test effects, or regression to the mean. However, regression to the mean may be a less plausible explanation as this study was designed to coincide with low-stress periods with respect to our study population. Further limitations include that effects have been estimated with low precision (although this is a limitation that characterizes almost all pilot studies). For these reasons, we must emphasize that all results should be treated as preliminary and for readers to avoid exaggerated generalizations of findings. Within the present study, the single-arm research design was an efficient use of limited resources to examine most uncertainties that may arise in each arm of a definitive trial, and to determine feasibility estimates for such a trial. Finally, it is important to note that the results of this study are mostly generalizable to nonclinical populations of young, healthy, and university educated adults.

## Conclusion

Retention rate and assessment response rate were acceptable, and a sample size analysis indicated that it is feasible to detect most effects in a definitive trial with precision. We recommend that a definitive randomized controlled trial is feasible and should proceed.

## Supplementary Information

**Additional file 1** Supplementary Materials.

**Additional file 2** Supplementary Results.

## Data Availability

The Supplementary Materials, Supplementary Results, CONSORT checklist, R statistical software analysis scripts, and instructions for reproducing all findings are available at the following GitHub repository: https://github.com/gprochilo/stress_trial. The deidentified data are available from the corresponding author upon a reasonable request. These data are not publicly available on the advice of the Monash Ethics Committee because the approved ethics application did not include provisions for public access to data.
