# Supplementary material for: A 16-week aerobic exercise and mindfulness-based intervention on chronic psychosocial stress: a pilot and feasibility study
Source: Pilot Feasibility Stud. 2021 Mar 6;7:64. doi: 10.1186/s40814-020-00751-6 (PMC7936438; doi:10.1186/s40814-020-00751-6)
Supplement: Supplementary file 1 — Additional file 1 Supplementary Materials. [file 40814_2020_751_MOESM1_ESM.pdf]

The Effects of a 16-week Aerobic Exercise and Mindfulness-based Intervention on Chronic Psychosocial Stress: A Pilot and Feasibility Study

Supplementary Materials

Guy A. Prochilo[1], Ricardo J.S. Costa[2], Craig Hassed[2], Richard Chambers[2], & Pascal Molenberghs[3]

[1] University of Melbourne
[2] Monash University
[3] Institute for Social Neuroscience

Guy A. Prochilo, Melbourne School of Psychological Sciences, University of Melbourne, Australia; Ricardo J.S. Costa, Department of Nutrition Dietetics & Food, Monash University; Craig Hassed, Department of General Practice, Monash University; Richard Chambers, Campus Community Division, Monash University; Pascal Molenberghs, Institute for Social Neuroscience.

Correspondence concerning this article should be addressed to Guy A. Prochilo, Melbourne School of Psychological Sciences, University of Melbourne. E-mail: guy.prochilo@gmail.com

Contents

Contents **2**

List of Tables **2**

Mindfulness-based intervention (MBI) **3**

Aerobic exercise training intervention (AET) **3**

Tables **5**

References **10**

List of Tables

1 Standard structure of the weekly training session 5
2 Training schedule and brief summary of each training session 6
3 Half-marathon training schedule 9

Mindfulness-based intervention (MBI)

The MBI was an adaptation of the mindfulness-based Stress Release Program (SRP; Hassed, 2002; Hassed, Lisle, Sullivan, & Pier, 2009): a stress management and cognitive therapy program that uses mindfulness practices to raise awareness of the processes underlying stress and negative emotions. The training approach can be summarized in two modules that align with the monitor and acceptance theory (MAT) of mindfulness meditation (Lindsay & Creswell, 2017). This includes: (1) attention monitoring, and (2) acceptance. The goal of the attention monitoring module was to systematically train participants to improve their ability to observe present-moment experiences through formal meditation practices (Lindsay & Creswell, 2017). The formal practice of meditation involved activities that required sustained focused-attention meditation towards a focus object (i.e., breath, body, sound, or thoughts). The formal practice prescription comprised up to 20 min of focused-attention meditation, daily, over 16 weeks. Formal practice was conducted in a group setting in a seminar room located at Monash University during the weekly training session, and participants were provided with audio recordings containing spoken instructions (Monash University Counselling Services) to guide home practice. To guide progress and track compliance, participants were further instructed to record their formal practice in an online spreadsheet (Google Sheets, Google, US). Formal practice was reported each day as minutes of formal meditation practice. This was converted to hours, and total hours of meditation for each participant across the intervention was recorded as the formal practice dosage. Adherence requirements for the attention monitoring module were completion of at least 50% of the target formal meditation (target = 37.34 h [20 min/day over 16-week]; adherence requirement = 18.67 h).

The goal of the acceptance module was to foster a mental attitude of acceptance towards negative and stress-laden experiences (Lindsay & Creswell, 2017). This included: (1) a willingness to allow negative experiences to rise and pass, and to let go of thoughts, ideas, or desires that may be associated with the experience (non-attachment), (2) a willingness to be available to negative experiences without attempting to suppress or avoid them (non-avoidance), and (3) a conscious abstention for categorizing an experience as good or bad, or right or wrong (non-judgment; Williams & Lynn, 2010). These skills were trained over eight weekly group training sessions, discussion activities, and homework tasks that encouraged participants to reflect on stress-laden or negative experiences with an attitude of acceptance (see Table 2). Adherence requirements for the acceptance module were that participants attend at least 50% of the group psychoeducation sessions, with compliance monitored through attendance records.

Aerobic exercise training intervention (AET)

The AET program comprised endurance running as part of a half-marathon training program. Endurance running was selected as the training modality because it relies predominantly on aerobic metabolism (Joyner & Coyle, 2008). Half-marathon training was selected because it includes a combination of moderate, vigorous, and high-intensity aerobic exercise intensities that each have potential to improve $\dot{V}O_{2max}$ and/or aerobic economy

(Bacon, Carter, Ogle, & Joyner, 2013; Bassett & Howley, 2000). The AET prescription was three runs per week over 16 weeks (one group session and two self-guided sessions). This included: (1) a 5 min warm up, then an interval run that involved 200 m bursts of near-maximal intensity followed by a 200 m recovery to the total of 16 min, then a 5 min cool down, (2) a short run of moderate or moderate-to-vigorous intensity that gradually increased in duration from 20 min to 60 min over 16 weeks, and (3) a long run at a moderate intensity or moderate-to-vigorous intensity that gradually increased in duration from 20 min to 110 min over 16 weeks. Additionally, participants completed three distance running trials at a moderate or moderate-to-vigorous intensity (Week 6 = 10 km, Week 9 = 10 km, Week 12 = 15 km). Exercise intensity categories were formally defined by the ACSM exercise prescription guidelines: (1) moderate intensity = 46 to < 64% $\dot{V}O_{2max}$, (2) vigorous intensity = 64 to < 91% $\dot{V}O_{2max}$, and (3) near-maximal intensity ≥ 91% $\dot{V}O_{2max}$ (Pescatello, Arena, Riebe, & Thompson, 2014). Running velocity corresponding to these intensity categories were individually prescribed to each participant by plotting oxygen consumption ($\dot{V}O_2$ mL/kg/min) against running velocity (km/h) in a regression model derived from a baseline $\dot{V}O_{2max}$ test. In this way, exercise intensity was completely individualized. The full half-marathon training schedule is described in Table 3. In addition to the AET, participants were asked to maintain their regular pattern of physical activity outside of the intervention (e.g., gym, leisure activities, and cycling commutes).

To guide performance and track compliance, participants were issued a computerized and GPS-enabled sportswatch (Garmin Forerunner 235, Garmin, US). This device provided participants real-time estimates of running velocity, and therefore of individual exercise intensity. Participants uploaded their running data to an online database (connect.garmin.com) weekly, which provided detailed information on training velocity, distance, and time. Adherence requirements for the AET program were completion of at least 50% of all prescribed runs (target = 48 individual runs [3 runs/week over 16 weeks]; adherence requirement = 24 runs). Exercise intensity was calculated for each running session for each participant using individual oxygen consumption ($\dot{V}O_2$ mL/kg/min) to running velocity (km/h) regression models, with the average running velocity of the running session as the velocity input. This was reported relative to participants' baseline $\dot{V}O_{2max}$ (i.e.,%$\dot{V}O_{2max}$ per session). The average exercise intensity of training across the full program was calculated as the mean of these values for each participant (i.e.,%$\dot{V}O_{2max}$ per intervention). Total running distance (km) and running time (h) were also recorded as measures of dosage.

Tables

Table 1

Standard structure of the weekly training session

Time (min)	Task
t_0	Feedback Session (group-based discussion on performance from the previous week of training; 15 min).
t_{0+15}	Training session content and group discussion exercises (35 min).
t_{0+50}	Guided meditation (audio-guided formal meditation practice; 10 min).
t_{0+60}	Intermission (changing clothes and travel to running track; 15 min).
t_{0+75}	Warm-up, running training, and warm-down activities (Up to 120 min).
t_{0+120}	End of session.

Table 2
Training schedule and brief summary of each training session

Week	Training Session	Summary
1	Formal and informal Meditation	Introduced participants to formal mindfulness practice. Systematic focused attention meditation was presented as the central therapeutic approach to the intervention. Participants were encouraged to use skills derived through formal practice in informal, everyday activities (e.g., mindfulness during everyday tasks, such as driving, eating, and cleaning).
2	Perception	Encouraged participants to use formal meditation during times of stress to raise present moment awareness, and to use this awareness to consider the event in a more objective, considered manner (i.e., is the stress response that is being experienced equivalent to the objective demands of the event?).
3	-	No training session.
4a	Cultivating Gentleness	Focused on fostering an attitude of acceptance to focused attention meditation. Early in mindfulness-based training participants can become self-critical frequent mind-wandering during mindfulness practice. Participants were instead encouraged to treat meditation as a learning experience, and to be gentle with themselves whenever their mind inevitably wanders during the practice.
4b	Letting Go	Encouraged participants to explore thoughts, ideas, desires, and expectations they were attached to, but which may be associated with stressful experiences. Participants were invited to consider what effect this may have on their experience of stress if we were to learn to let go (i.e., practice non-attachment).
5a	Letting Go (cont.)	See above.

Table 2 continued

Week	Training Session	Summary
5b	Acceptance	Encouraged participants to explore acceptance of: (1) internal (e.g., emotions) and external experiences (e.g., the actions of others), and (2) the responsibility to make changes that are within one's power (e.g., if stress is caused by physical inactivity, to accept the responsibility it will take to become more active).
6	10 km Trial I	A running-only training session that comprised a 10km trial ran at a comfortable pace.
7	-	No training session.
8	Preparing for Long-distance Training	Covered nutrition requirements necessary for (pre-, post-, and during-) long-distance running training to optimize performance and reduce risk of problems while running. Also examined clothing requirements and strategies for completing longer runs.
9	10 km Trial II	A running-only training session that comprised a 10km trial. Participants were encouraged to improve their Trial I performance.
10	Presence of Mind	Focused on discussion and reflection activities that made use of mindfulness in everyday activities (i.e., the informal practice of mindfulness). Discussion topics included how stress can be linked to a lack of mindfulness in these everyday tasks (e.g., through multitasking, unmindful communication, and the difference between worry and planning).
11	Improving Fitness	Covered information on how to maximize improvements in aerobic fitness based on the ACSM's exercise prescription guidelines.
12	15 km Trial	A running-only training session that comprised a 15km trial completed at a comfortable pace.
13	Avoiding Injuries	Covered strategies and core strength exercises with a focus on prevention of common running injuries. General strategies for overcoming present injuries were also provided.

Table 2 continued

Week	Training Session	Summary
14	Mindfulness and Negative Emotions I	This session was designed to draw together all of the mindfulness training concepts and to apply them specifically to commonly held mindsets relating to negative emotions. The first training session focused on stress mindsets, how to let go of a 'stress is debilitating' mindset through mindfulness (e.g., how to accept that stress is a part of everyday life, and how stress may even be a resource). The second training session covered similar content, but instead focused on sadness, and letting go of a sadness-avoidance mindset (e.g., accepting that sad emotions are part of the tapestry of normal human emotions, and there is no need to pathologize negative emotions).
15	Mindfulness and Negative Emotions II	See above.
16	Review	A review session of the mindfulness-based program (attention monitoring and acceptance modules) and the running training support module. Also, a final review of mindfulness and aerobic training progress, and a series of best-practice guidelines to safely complete an optional half-marathon.

Table 3
Half-marathon training schedule

Week	Group Session	Beginner Tier	Intermediate Tier
1	5 km easy	S: 20 min easy<br>L: 20 min easy	S: 30 min easy<br>L: 45 min easy
2	I: 16 min intervals	S: 20 min easy<br>L: 20 min easy	S: 40 min easy<br>L: 45 min easy
3	I: 16 min intervals	S: 20 min steady<br>L: 20 min steady	S: 45 min steady<br>L: 60 min easy
4	I: 16 min intervals	S: 20 min steady<br>L: 20 min steady	S: 50 min steady<br>L: 65 min easy
5	I: 16 min intervals	S: 20 min steady<br>L: 35 min steady	S: 50 min easy<br>L: 55 min steady
6	10 km trial I	S: 20 min easy<br>L: 30 min steady	S: 20 min easy<br>L: 50 min steady
7	I: 16 min intervals	S: 40 min steady<br>L: 50 min easy	S: 55 min steady<br>L: 75 min easy
8 (T)	I: 16 min intervals	S: 20 min easy<br>L: 20 min easy	T: 30 min easy<br>T: 30 min easy
9	10 km trial II	S: 35 min steady<br>I: 16 min intervals	S: 35 min steady<br>I: 16 min intervals
10	I: 45 min easy	S: 45 min easy<br>L: 75 min easy	S: 45 min steady<br>L: 60 min steady
11	I: 16 min intervals	S: 40 min steady<br>L: 60 min easy	S: 50 min steady<br>L: 65 min steady
12	15 km trial	S: 20 min easy<br>L: 55 min steady	S: 20 min easy<br>L: 55 min steady
13	I: 16 min intervals	S: 50 min steady<br>L: 100 min easy	S: 60 min steady<br>L: 100 min easy
14	I: 16 min intervals	S: 50 min steady<br>L: 110 min easy	S: 50 min steady<br>L: 110 min easy
15 (T)	T: 50 min easy	T: 50 min easy<br>T: 50 min easy	T: 50 min easy<br>T: 50 min easy
16	T: 20 min steady	T: 20 min steady<br>T: 20 min steady	T: 20 min steady<br>T: 20 min steady

Note. All participants were asked to run three times per week: one group session (identical for both training tiers) and two additional runs completed in their own time and location of preference. All runs began with 5 – 10 min of warm-up (slow run and dynamic warm-up movements). I = interval run; S = short run; L = long run; D = distance trial, T = taper period, easy = moderate intensity (moderate = 46 to 64% $\dot{V}O_{2max}$), steady = constant pace at moderate to vigorous intensity (vigorous = 64 to 91% $\dot{V}O_{2max}$), intervals = 200 m bursts at near maximal intensity ($\geq$ 91% $\dot{V}O_{2max}$) followed by 200 m recovery; taper = period of less activity to recover from training or prepare for distance events.

References

Bacon, A. P., Carter, R. E., Ogle, E. A., & Joyner, M. J. (2013). VO(2)max trainability and high intensity interval training in humans: A meta-analysis. *PLoS ONE*, *8*(9), e73182. doi: 10.1371/journal.pone.0073182

Bassett, J., D. R., & Howley, E. T. (2000). Limiting factors for maximum oxygen uptake and determinants of endurance performance. *Med Sci Sports Exerc*, *32*(1), 70–84.

Hassed, C. (2002). *Know thyself: The stress release programme.* Australia: McPhersons Printing.

Hassed, C., Lisle, S. de, Sullivan, G., & Pier, C. (2009). Enhancing the health of medical students: Outcomes of an integrated mindfulness and lifestyle program. *Adv Health Sci Educ Theory Pract*, *14*(3), 387–398. doi: 10.1007/s10459-008-9125-3

Joyner, M. J., & Coyle, E. F. (2008). Endurance exercise performance: The physiology of champions. *The Journal of Physiology*, *586*(1), 35–44. doi: doi:10.1113/jphysiol.2007.143834

Lindsay, E. K., & Creswell, J. D. (2017). Mechanisms of mindfulness training: Monitor and acceptance theory (mat). *Clinical Psychology Review*, *51*, 48–59. doi: 10.1016/j.cpr.2016.10.011

Pescatello, L. S., Arena, R., Riebe, D., & Thompson, P. D. (2014). *ACSM's guidelines for exercise testing and prescription* (9th ed.). Philadelphia: Wolters Kluwer/Lippincott Williams & Wilkins Health.
