# Supplementary material for: A 16-week aerobic exercise and mindfulness-based intervention on chronic psychosocial stress: a pilot and feasibility study
Source: Pilot Feasibility Stud. 2021 Mar 6;7:64. doi: 10.1186/s40814-020-00751-6 (PMC7936438; doi:10.1186/s40814-020-00751-6)
Supplement: Supplementary file 2 — Additional file 2 Supplementary Results. [file 40814_2020_751_MOESM2_ESM.pdf]

Supplementary Results

The Effects of a 16-week Aerobic Exercise and Mindfulness-based Intervention on Chronic Psychosocial Stress: A Pilot and Feasibility Study

Guy A. Prochilo[1], Ricardo J.S. Costa[2], Craig Hassed[2], Richard Chambers[2], & Pascal Molenberghs[3]

[1] University of Melbourne
[2] Monash University
[3] Institute for Social Neuroscience

Guy A. Prochilo, Melbourne School of Psychological Sciences, University of Melbourne, Australia; Ricardo J.S. Costa, Department of Nutrition Dietetics & Food, Monash University; Craig Hassed, Department of General Practice, Monash University; Richard Chambers, Campus Community Division, Monash University; Pascal Molenberghs, Institute for Social Neuroscience.

Correspondence concerning this article should be addressed to Guy A. Prochilo, Melbourne School of Psychological Sciences, University of Melbourne. E-mail: guy.prochilo@gmail.com

List of Tables

1 Summarized results of the linear mixed model for absolute oxygen cost. . . 3
2 Summarized results of the linear mixed model for relative oxygen cost. . . . 4
3 Summarized results of the linear mixed model for heart rate. 5
4 Summarized results of the linear mixed model for perceived exertion (RPE). 6

Table 1

Summarized results of the linear mixed model for absolute oxygen cost.

Random Effects	Log Likelihood	AIC	LRT	*df*	*p*
Participant	-38.17	96.34			
None	-78.74	175.48	81.14	1	<.001
	Estimate	*SE*	*df*	*t*	*p*
Intercept	2.10	0.11	12.00	18.90	<.001
Time1	0.07	0.02	84.00	2.75	.007
Velocity1	-0.90	0.04	84.00	-20.88	<.001
Velocity2	-0.20	0.04	84.00	-4.69	<.001
Velocity3	0.33	0.04	84.00	7.69	<.001
Time1 x Velocity1	-0.05	0.04	84.00	-1.28	.205
Time1 x Velocity2	-0.03	0.04	84.00	-0.79	.430
Time1 x Velocity3	0.03	0.04	84.00	0.60	.552
	Variance	*SD*			
Random effects	0.15	0.39			
Residual	0.06	0.25			

Note. Sampling Units: N total observations = 104; N participants = 13. Final model equation: $oxygen\ cost \sim time \times velocity + (1|participant)$; t statistics and p values calculated using Satterthwaite's method; AIC = Akaike Information Criterion; LRT = Likelihood Ratio Test; df = degrees of freedom; p = p value; SD = standard deviation. Factors: Time1 = T0, Velocity1 = 6 km/h, Velocity2 = 8 km/h, Velocity3 = 10 km/h.

Table 2

Summarized results of the linear mixed model for relative oxygen cost.

Random Effects	Log Likelihood	AIC	LRT	*df*	*p*
Participant	-327.68	675.36			
None	-341.19	700.38	27.02	1	<.001
	Estimate	*SE*	*df*	*t*	*p*
Intercept	61.31	1.37	12.00	44.66	<.001
Time1	2.18	0.54	84.00	4.01	<.001
Velocity1	-26.54	0.94	84.00	-28.14	<.001
Velocity2	-6.12	0.94	84.00	-6.49	<.001
Velocity3	9.86	0.94	84.00	10.46	<.001
Time1 x Velocity1	-1.48	0.94	84.00	-1.56	.121
Time1 x Velocity2	-1.02	0.94	84.00	-1.08	.284
Time1 x Velocity3	0.77	0.94	84.00	0.82	.417
	Variance	*SD*			
Random effects	20.65	4.54			
Residual	30.82	5.55			

Note. Sampling Units: *N* total observations = 104; *N* participants = 13. Final model equation: $relative\ oxygen\ cost \sim time \times velocity + (1|participant)$; *t* statistics and *p* values calculated using Satterthwaite's method; AIC = Akaike Information Criterion; LRT = Likelihood Ratio Test; *df* = degrees of freedom; *p* = *p* value; SD = standard deviation. Factors: Time1 = T0, Velocity1 = 6 km/h, Velocity2 = 8 km/h, Velocity3 = 10 km/h.

Table 3

Summarized results of the linear mixed model for heart rate.

Random Effects	Log Likelihood	AIC	LRT	*df*	*p*
Participant	-369.37	758.75			
None	-399.71	817.42	60.67	1	<.001
	Estimate	*SE*	*df*	*t*	*p*
Intercept	154.93	2.99	12.00	51.89	<.001
Time1	2.28	0.80	84.00	2.85	.005
Velocity1	-31.73	1.39	84.00	-22.89	<.001
Velocity2	-5.78	1.39	84.00	-4.17	<.001
Velocity3	12.07	1.39	84.00	8.70	<.001
Time1 x Velocity1	-0.94	1.39	84.00	-0.68	.498
Time1 x Velocity2	-0.13	1.39	84.00	-0.09	.926
Time1 x Velocity3	0.03	1.39	84.00	0.02	.982
	Variance	*SD*			
Random effects	107.56	10.37			
Residual	66.62	8.16			

Note. Sampling Units: N total observations = 104; N participants = 13. Final model equation: $heart\ rate \sim time \times velocity + (1|participant)$; t statistics and p values calculated using Satterthwaite's method; AIC = Akaike Information Criterion; LRT = Likelihood Ratio Test; df = degrees of freedom; p = p value; SD = standard deviation. Factors: Time1 = T0, Velocity1 = 6 km/h, Velocity2 = 8 km/h, Velocity3 = 10 km/h.

Table 4

Summarized results of the linear mixed model for perceived exertion (RPE).

Random Effects	Log Likelihood	AIC	LRT	*df*	*p*
Participant	-160.46	340.92			
None	-174.08	366.16	27.24	1	<.001
	Estimate	*SE*	*df*	*t*	*p*
Intercept	10.91	0.24	12.00	45.25	<.001
Time1	0.36	0.10	84.00	3.73	<.001
Velocity1	-3.53	0.17	84.00	-21.37	<.001
Velocity2	-0.91	0.17	84.00	-5.53	<.001
Velocity3	1.05	0.17	84.00	6.35	<.001
Time1 x Velocity1	-0.20	0.17	84.00	-1.22	.225
Time1 x Velocity2	0.11	0.17	84.00	0.64	.524
Time1 x Velocity3	0.07	0.17	84.00	0.41	.685
	Variance	*SD*			
Random effects	0.64	0.80			
Residual	0.95	0.97			

Note. Sampling Units: N total observations = 104; N participants = 13. Final model equation: $RPE \sim time \times velocity + (1|participant)$; t statistics and p values calculated using Satterthwaite's method; AIC = Akaike Information Criterion; LRT = Likelihood Ratio Test; *df* = degrees of freedom; p = p value; SD = standard deviation. Factors: Time1 = T0, Velocity1 = 6 km/h, Velocity2 = 8 km/h, Velocity3 = 10 km/h.
